# The utility of whole-genome sequencing to inform epidemiologic investigations of SARS-CoV-2 clusters in acute-care hospitals

**DOI:** 10.1017/ice.2023.274

**Published:** 2024-02

**Authors:** Theodore S. Rader, Vatsala R. Srinivasa, Marissa P. Griffith, Kady Waggle, Lora Pless, Ashley Chung, Suzanne Wagester, Lee H. Harrison, Graham M. Snyder

**Affiliations:** 1 Division of Infectious Diseases, University of Pittsburgh School of Medicine, Pittsburgh, Pennsylvania; 2 Microbial Genomics Epidemiology Laboratory, Center for Genomic Epidemiology, University of Pittsburgh, Pittsburgh, Pennsylvania; 3 Wolff Center, UPMC, Pittsburgh, Pennsylvania; 4 Department of Infection Prevention and Control, UPMC Presbyterian/Shadyside, Pittsburgh, Pennsylvania

## Abstract

**Objective:**

To evaluate the utility of selective reactive whole-genome sequencing (WGS) in aiding healthcare-associated cluster investigations.

**Design:**

Mixed-methods quality-improvement study.

**Setting:**

Thes study was conducted across 8 acute-care facilities in an integrated health system.

**Methods:**

We analyzed healthcare-associated coronavirus disease 2019 (COVID-19) clusters between May 2020 and July 2022 for which facility infection prevention and control (IPC) teams selectively requested reactive WGS to aid the epidemiologic investigation. WGS was performed with real-time results provided to IPC teams, including genetic relatedness of sequenced isolates. We conducted structured interviews with IPC teams on the informativeness of WGS for transmission investigation and prevention.

**Results:**

In total, 8 IPC teams requested WGS to aid the investigation of 17 COVID-19 clusters comprising 226 cases and 116 (51%) sequenced isolates. Of these, 16 (94%) clusters had at least 1 WGS-defined transmission event. IPC teams hypothesized transmission pathways in 14 (82%) of 17 clusters and used data visualizations to characterize these pathways in 11 clusters (65%). The teams reported that in 15 clusters (88%), WGS identified a transmission pathway; the WGS-defined pathway was not one that was predicted by epidemiologic investigation in 7 clusters (41%). WGS changed the understanding of severe acute respiratory syndrome coronavirus 2 (SARS-CoV-2) transmission in 8 clusters (47%) and altered infection prevention interventions in 8 clusters (47%).

**Conclusions:**

Selectively utilizing reactive WGS helped identify cryptic SARS-CoV-2 transmission pathways and frequently changed the understanding and response to SARS-CoV-2 outbreaks. Until WGS is widely adopted, a selective reactive WGS approach may be highly impactful in response to healthcare-associated cluster investigations.

Healthcare-associated infections due to severe acute respiratory syndrome coronavirus 2 (SARS-CoV-2) represent a preventable risk to patients and healthcare workers. Hospital-onset coronavirus disease 2019 (COVID-19) cases have been associated with increased length of stay and higher mortality.^1,2^ Identifying and interrupting SARS-CoV-2 transmission in acute-care settings can be challenging due to the incubation period,^3^ contagiousness in asymptomatic individuals,^4^ community infection prevalence levels,^5^ and variable compliance with infection prevention measures.^6,7^ These characteristics make SARS-CoV-2 an apt model to investigate the role of using genetic relatedness testing to confirm potential transmission routes identified by epidemiologic investigation.

Whole-genome sequencing (WGS) is currently the gold standard tool for elucidating in-hospital transmission pathways in acute-care settings.^8–12^ For COVID-19 disease, approaches have included both WGS of all hospital-onset isolates (“WGS surveillance”) to identify transmission events and reactive sequencing to investigate potential outbreaks.^2,13–15^ WGS surveillance identifies genetically related viruses in individuals without epidemiologic links in as many as 11%–22% of sequenced specimens.^8^ Although WGS surveillance may identify all genetically related COVID-19 cases, it has practical limitations including resource costs, experienced staff, and availability.^16,17^

Reactive sequencing methods utilize WGS to confirm or refute hypothesized transmission routes after a suspected outbreak to provide useful information in identifying a cluster and performing an epidemiologic investigation.^8,9^ Where surveillance WGS is unfeasible and there are barriers to the routine use of reactive WGS, a selective reactive strategy may be appropriate. Guidance on when and how to employ the selective use of reactive WGS for infection prevention is mostly limited to potential use cases with at least 1 suggested clinical decision aid,^18^ although at the time of writing this approach had not been applied to COVID-19.^19^ Understanding when reactive WGS is most impactful may help inform effective use of a limited resource for COVID-19 and potentially other healthcare-associated pathogens.

In this quality improvement evaluation, we retrospectively reviewed the utility of selective reactive WGS to aid COVID-19 cluster investigations in a multifacility health system. We present a description of COVID-19 clusters, genomic findings, and an interview-based mixed-methods examination of the impact of selective reactive WGS to elucidate transmission pathways and inform infection prevention responses.

## Methods

### Setting and design

UPMC is a 40-hospital, integrated academic healthcare system with coordinated infection prevention practices.^20^ Individual facility infection prevention and control (IPC) teams facilitate COVID-19 contact tracing, conduct cluster investigations, and perform public health reporting. The characteristics of facilities included in this analysis that used WGS to support at least 1 COVID-19 outbreak investigation are described in Supplementary Table S1 (online). Admission screening was not performed for asymptomatic individuals except for facility F, an acute-care behavioral health hospital. This study includes outbreaks that were investigated from May 2020 through July 2022.

We conducted a mixed-methods study to understand the impact of WGS to elucidate transmission pathways and inform IPC responses.^21^ The quantitative phase consisted of characterizing findings from selective WGS used by IPC teams in their epidemiological investigations of potential healthcare-associated COVID-19 outbreaks. The qualitative phase utilized structured interviews with IPC teams to explore the contribution of WGS to COVID-19 cluster investigations and impact on IPC team practices.

This investigation underwent formal review and was granted ethical approval (project nos. 4092 and 4094) as a quality improvement project by the UPMC Quality Improvement Review Committee.

### Genomic relatedness of SARS-CoV-2 isolates

Reactive WGS is performed to support epidemiologic cluster investigations at the Microbial Genomics Epidemiology Laboratory (MiGEL) at the University of Pittsburgh.^22,23^ Reactive WGS was available as a resource for COVID-19 cluster investigations within UPMC facilities through a structured request process (Supplementary Fig. S1 online). Local IPC teams reviewed all SARS-CoV-2-positive tests among patients and reported illness or positive tests among healthcare workers. They also performed contact tracing to identify potentially exposed individuals. Patients and healthcare workers were considered a case at the discretion of investigating local IPC team based on nucleic acid amplification testing, antigen testing, and/or COVID-19 disease based on epidemiologic exposure. If a potential cluster was identified, local IPC investigated possible source(s) and transmission routes and then implemented or reinforced infection prevention measures. Asymptomatic screening was routinely performed among patients following an exposure identified via contact tracing. Asymptomatic screening was selectively and infrequently used among healthcare workers and/or indirect unit-based patient contacts when an outbreak was suspected but transmission pathways could not be ascertained. IPC teams could request WGS of cluster isolates to resolve uncertain transmission pathways or understand failures of IPC practice to inform future prevention measures.

Clinical nasal or nasopharyngeal swab samples sequenced in this study were obtained from Food and Drug Administration-approved nucleic acid amplification testing platforms or molecular laboratory developed test [ref: https://www.fda.gov/media/140545/download]. These isolates were collected by MiGEL and were deidentified for sequencing. Nucleic acids were extracted using the MagMAX Viral RNA/Pathogen isolation kits (ThermoFisher Scientific, Waltham, MA) according to the manufacturer’s instructions. Sequencing libraries were prepared using either the ARTIC V3 protocol^24^ or the Illumina RNA prep with enrichment (L) protocol and the respiratory virus oligo panel (RVOPv1).^25^ Libraries were sequenced on a NextSeq550 high-output flow cell using 150-bp paired-end reads. The resulting reads were aligned to Wuhan-Hu-1 (MN908947) reference sequence. A detailed description of the genomic data analyses is presented in Srinivasa et al.^22^ Briefly, single-nucleotide polymorphisms (SNPs) were identified using Breseq and hierarchical clustering was performed using the single linkage clustering method for all clusters except cluster 14. For cluster 14, average linkage clustering with a 3-SNP cutoff was used. A pairwise SNP difference of ≤2 was used to define genetically related SARS-CoV-2 genomes for all other clusters.^17^

For each request, a report was prepared that included a pairwise SNP distance matrix, Pangolin lineages for sequenced isolates, and a detailed explanation of the genomic investigation. The report was provided to the requesting local IPC teams and to UPMC system IPC leadership to augment the traditional epidemiologic investigation.

### Structured interviews

Structured interviews of local IPC teams were conducted in January and February of 2023 separately for each individual cluster for which WGS was performed. Interviews were conducted by one investigator (T.R.) using a standardized interview form (Supplementary Fig. S2 online). Prior to the interview, additional IPC materials were requested (if present) to supplement understanding of cluster investigations, including line lists, transmission visualizations, email communications, and other pertinent investigation documentation. Extended responses were recorded as field notes that were discussed with the IPC team to ensure agreement with qualitative statements. These responses underwent inductive coding to identify common themes for reporting (by T.R.). Quantitative and qualitative components from the interviews were reported as frequencies. Interviews were conducted and data were recorded using an organization-hosted web-based application suite (Microsoft Teams, Forms and Excel; Redmond, WA). Consolidated criteria for Reporting Qualitative research (COREQ) framework was used to enhance reporting of structured interview responses.^26^

## Results

### Description of investigated clusters

Between May 1, 2020, and August 1, 2022, IPC teams from 8 UPMC facilities requested and received reactive WGS analyses for 17 COVID-19 clusters. These clusters comprised 226 adults identified as potentially part of an outbreak: 132 patients (58%) and 94 healthcare workers (24%). The median number of individuals in the suspected outbreaks was 10 (range, 3–26). Of 226 suspected cases, 182 isolates (81%) were submitted for sequencing, from which 116 high-quality genomes (51%) were obtained for cluster analysis (Supplementary Tables S2 and S3 online).

### Genomic characterization of SARS-CoV-2 outbreak isolates

**Figure 1.**
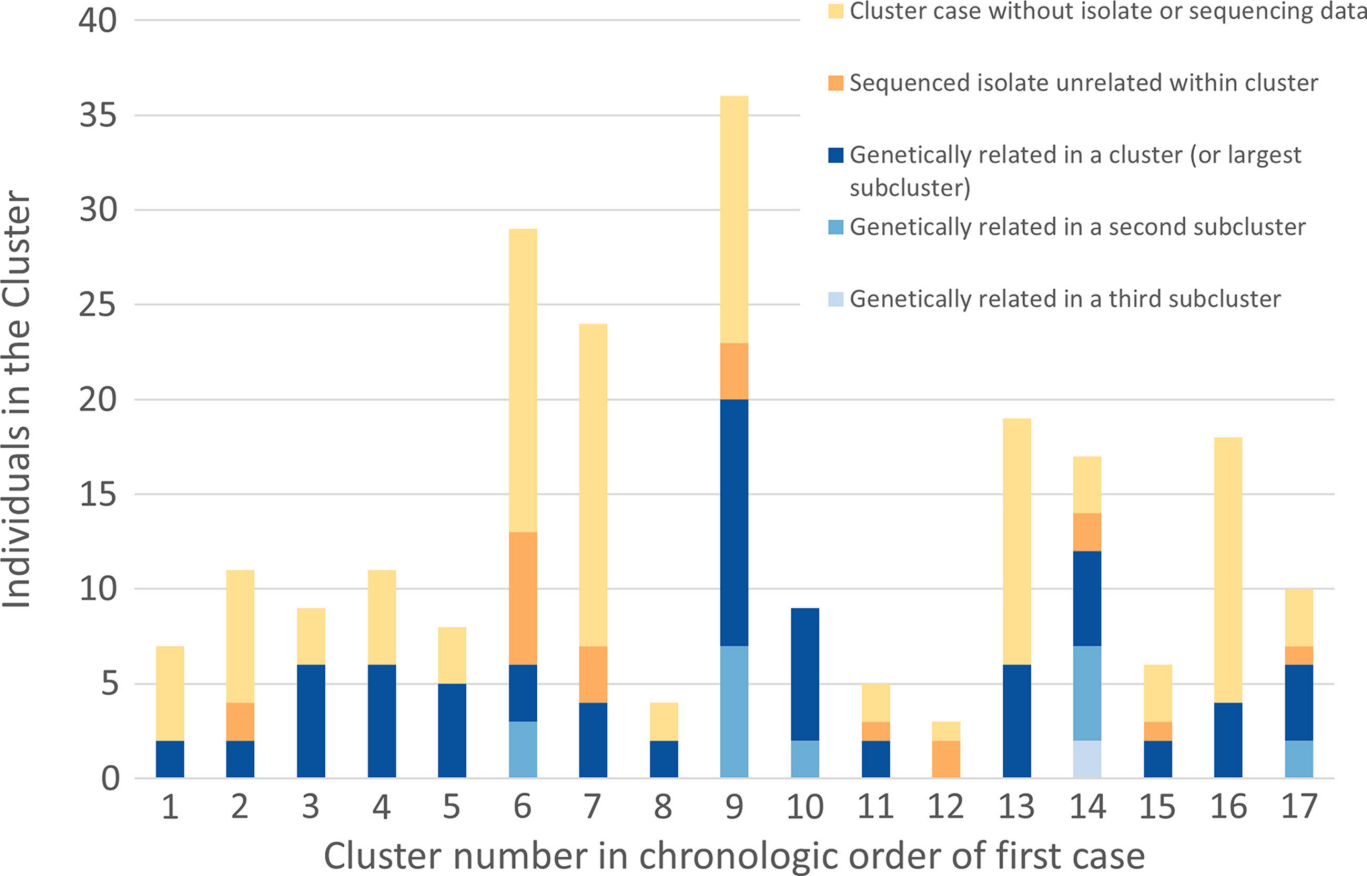
Genetic relatedness among SARS-CoV-2 isolates within investigated healthcare-associated COVID-19 clusters.

**Figure 2.**
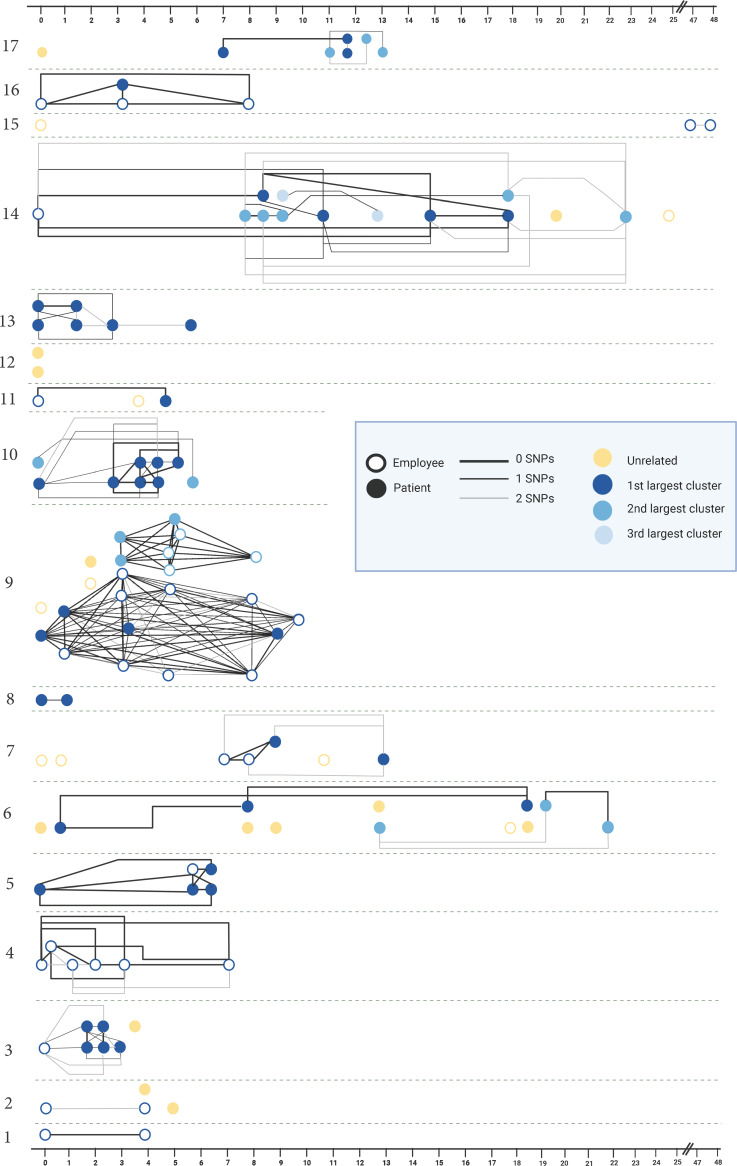
Genomic clustering of SARS-CoV-2 isolates among the 17 investigated clusters. Note: SNP, single-nucleotide polymorphism; x-axis denotes days since initial case in the cluster.

Among 17 investigated clusters, 16 clusters (94%) had ≥2 genetically related isolates among sequenced isolates (Fig. 1). Also, 5 clusters (29%) had multiple genetically distinct subclusters, including 4 (24%) with 2 subclusters (clusters 6, 9, 10 and 17) and 1 cluster (6%) with 3 subclusters (cluster 14). Figure 2 shows the genomic clustering of sequenced SARS-CoV-2 isolates among the 16 investigated clusters with ≥2 genetically related isolates. The average number of genetically related isolates within an investigated cluster was 5.5 (range, 2–20). A heatmap of the pairwise SNP comparisons is provided in Supplementary Figure 3 (online).

When we compared the SARS-CoV-2 genomes used in the study to publicly available genomes from Pennsylvania, the study isolates represented circulating strains in the community at the time of each cluster (data not shown, sequence information in Supplementary Materials online).

### Structured interviews

Overall, 17 structured interviews were completed among the 8 IPC teams requesting WGS to support cluster investigations. One IPC team supports both facilities A and H as well as 2 separate teams support the acute-care and long-term care clinical areas of facility D. Details of cluster investigations, including epidemiologically suspected transmission routes, WGS-supported transmission routes, and description of the clusters are provided in Supplementary Tables S4a and S4b (online). The most common suspected transmission routes on epidemiologic investigation by IPC teams prior to WGS were patient-to-patient and healthcare worker-to-patient routes, each occurring in 53% of investigations. The most common WGS-supported transmission route was patient to patient (53%), followed by healthcare worker to patient (41%).

Transmission pathways were hypothesized prior to WGS in 82% of cluster investigations, and transmission visualizations were used in ∼65% of investigations (Table 1). When transmission visualizations were used, 10 were Gantt charts (clusters 1, 3, 4, 5, 6, 7, 11, 12, 14 and 15), 2 were spider charts (clusters 9 and 16), 1 was a cluster map (cluster 7), and 1 was a timeline (cluster 4). Selected examples of data visualizations are provided in Supplementary Figures S4a–S4c (online).

**Table 1.** Results of Infection Prevention and Control Team Structured Interviews of COVID-19 Cluster Investigations Supported by Whole-Genome Sequencing

Survey Question	Response Frequency (%)		
	Yes	No	Unknown/Not Applicable
**Transmission pathway generation**			
Was/were there hypothesized transmission pathway(s) before the WGS request was placed?	14 (82)	2 (12)	1 (6)
Was there a transmission visualization of the outbreak?	11 (65)	6 (35)	0
**Interpretation of WGS results**			
Did WGS cluster analysis of this outbreak help identify a definitive pathway(s)?	15 (88)	2 (12)	0
Was [the WGS-supported pathway] one of your hypothesized pathways?	8 (47)	7 (41)	2 (12)
Did the WGS information change your understanding of the SARS-CoV-2 nosocomial transmission during the outbreak in question…?	8 (47)	8 (47)	1 (6)
**Impact of WGS results on infection prevention practices**			
Did the WGS information from this cluster change your interventions to interrupt SARS-CoV-2 transmission at the time you received it?	8 (47)	8 (47)	1 (6)

Note: WGS, whole-genome sequencing

IPC teams reported that WGS identified at least 1 transmission pathway in 16 (88%) of 17 clusters. The WGS-identified pathway was not a predicted pathway in 7 clusters (41%), and WGS changed the understanding of transmission in 8 clusters (47%) (Table 1). IPC teams reported changing the interventions to prevent further transmission in 8 (47%) of 17 cases (Table 1). Examples of changes included the following: education of relevant stakeholders (8 clusters), enhanced cleaning procedures (4 clusters), and changed the use of common spaces (2 clusters). In the 8 cluster investigations for which WGS results changed the understanding of transmission, all 8 investigations (100%) had a pre-WGS hypothesized pathway and 7 (88%) used transmission visualizations. For the 8 investigations for which infection prevention measures were changed because of WGS, 7 (88%) had pre-WGS hypothesized transmission pathways and 6 (75%) used transmission visualizations.

## Discussion

In this mixed-methods analysis of 17 COVID-19 clusters in 8 healthcare facilities for which reactive WGS was used, 16 (94%) of the clusters had 1 or more WGS-defined outbreaks. WGS provided likely transmission pathways in 88% of suspected outbreaks, revealing a novel pathway or elucidating transmission pathways in >40% of the investigated clusters, and affecting a change in the interventions to interrupt transmission. Using COVID-19 as a model, we have demonstrated the utility of selective reactive WGS.

We found that outbreaks were not frequently resolvable using traditional epidemiologic methods alone, which was consistent with multiple studies implicating the effectiveness of WGS in both confirming and refuting cryptic transmission.^8,27–29^ Adding WGS can identify transmission events that may be either missed or misidentified using traditional epidemiologic methods. In the COG-UK study, investigators performed prospective sequencing and provided either “rapid” (<48 hours) or “longer-turnaround” (5–10 days) feedback to IPC teams to assist in cluster investigations and transmission interruption.^17^ The intervention was resource intensive, returned reports in <50% of cases, and did not demonstrate a decrease in hospital-onset COVID-19 compared to a baseline period without sequencing. However, the information did change IPC interventions in ∼7%–20% of cases.^17^ Although our study was not designed to test the impact of WGS in reducing transmission, we did demonstrate that selective reactive WGS can be effective with less resource consumption and that it retains potential to reduce future transmission events.

Our study is not the first to show the utility of a reactive WGS strategy in COVID-19 infection prevention. In a single-center, 18-month trial using “on-demand” reactive WGS to characterize 6 outbreaks, WGS commonly refuted epidemiologic hypotheses for transmission (29% of outbreaks) and informed infection prevention measures, changing practice in 5 of 6 (83%) outbreaks.^30^ However, this approach was not selective because it used defined criteria to trigger investigations of outbreaks with WGS, and did not describe epidemiologic investigation characteristics that may have indicated where WGS was most useful. In our investigation, we attempted to characterize the hypothesis generation about transmission pathways that preceded requests for WGS. Nearly all teams generated hypotheses to be tested by WGS and ∼65% utilized transmission visualizations to assist.^31^ Where WGS changed transmission understanding or prevention measures, IPC teams frequently hypothesized pathways and used visualizations. This robust hypothesis generation, and the high frequency of genomic relatedness in our clusters, meant that we were unable to assess whether selective reactive WGS has utility in less well characterized cluster investigations. If a resource-sparing, selective WGS approach could be successful in reducing transmission risk, while reducing the costs of WGS investigation of outbreaks for COVID-19 or other pathogens, future studies will need to further elaborate on how infection preventionists identify and perform preliminary investigation of clusters for which WGS will be most informative.

This study had several limitations. First, the use of reactive WGS was not randomly selected and the investigation was not designed as a controlled trial of selective reactive WGS. Second, the utility of reactive WGS to a heterogenous group of experienced and qualified IPC team members in consultation with IPC leadership may not be generalizable to all healthcare settings. Third, structured interviews were conducted retrospectively, and interview results could have been affected by staff attrition and loss of investigation documentation, potentially diminishing our understanding of response to WGS results. This potential missing information may have resulted in an overestimation of the impact of WGS. Fourth, WGS of isolates was subject to availability. Isolate sequencing was not performed for ∼50% of the individuals epidemiologically identified in the investigated clusters. These isolates may not be missing randomly (eg, insufficient viral titer and genetic sequence may be correlated with transmission risk), and nonsequenced isolates could have yielded undetected or unexpected transmission pathways. Finally, we were unable to directly compare the costs of our approach compared to no use of WGS or WGS surveillance because the cost estimate was beyond the scope of this work.^16,32^

In this study, we demonstrated an approach to a selective use of reactive WGS for healthcare-associated COVID-19 cluster investigation. We prioritized a priori transmission pathway hypothesis generation with or without data visualization, which can yield a high likelihood of WGS informativeness. This approach changed our understanding of transmission pathways and modified IPC interventions for COVID-19 clusters. Until real-time WGS surveillance is widely available and adopted, a selective approach to reactive WGS is an effective and cost-efficient tool to assist in the investigation of COVID-19 outbreaks in the hospital.

## Supplementary material

For supplementary material accompanying this paper visit https://doi.org/10.1017/ice.2023.274. click here to view supplementary material
