# Supplementary material for: The utility of whole-genome sequencing to inform epidemiologic investigations of SARS-CoV-2 clusters in acute-care hospitals
Source: Infect Control Hosp Epidemiol. 2023 Dec 22;45(2):144–9. doi: 10.1017/ice.2023.274 (PMC10877536; doi:10.1017/ice.2023.274)
Supplement: Supplementary file 1 [file S0899823X2300274Xsup.zip › S0899823X2300274Xsup002.pdf]

Supplemental Materials

The Utility of Whole Genome Sequencing to Inform Epidemiologic Investigations of SARS-CoV-2 Clusters in Acute Care Hospitals: A Quality Improvement Evaluation
Rader T., et al.

Table of Contents

Table or Figure	Page #
1. Supplemental Table S1. Characteristics of facilities using whole genome sequencing to support COVID-19 cluster investigations	2
2. Supplemental Figure S1. UPMC system-level process for identifying, requesting, and reporting results of clusters investigated using whole genome sequencing	3
3. Supplemental Figure S2. Structured interview tool to assess infection prevention & control teams' use of whole genome sequencing to support COVID-19 cluster investigation	4
4. Supplemental Table S2. Information cascade from cases suspected in a healthcare-associated COVID-19 cluster to isolates with whole genome sequencing available	5
5. Supplemental Table S3: Sequence information for SARS-CoV-2 genomes included in this study	6
6. Supplemental Figure S3: Pairwise single nucleotide polymorphism comparison for healthcare-associated SARS-CoV-2 isolates identified in the 17 healthcare-associated clusters	9
7. Supplemental Tables S4a and S4b. Epidemiologic findings of COVID-19 cluster investigations in eight healthcare facilities	10
8. Supplemental Figures S4a-S4c: Examples of data visualizations used to hypothesize transmission pathways in COVID-19 cluster investigations	15

Supplemental Table S1. Characteristics of facilities using whole genome sequencing to support COVID-19 cluster investigations. Hospitals are for adult care, with exception of Hospital F, which has both adult and pediatric psychiatric beds although no pediatric patients were part of the study.

Facility	Facility type	Inpatient bed count						
		Intensive care unit	Medical/surgical	Step-down	Rehabilitation	Clinical decision unit	Behavioral health	Total
Hospital A	Acute care hospital	133	382	60	20			595
Hospital B	Acute care hospital	47	148	23	60	12	18	308
Hospital C	Community hospital	16	100	19	14	13		162
Hospital D	Community hospital with long-term care facility	6	71		11			88
Hospital E	Community hospital	10	123	24	22			179
Hospital F	Acute care behavioral health hospital						192	192
Hospital G	Community hospital		48	13	9		60	130
Hospital H	Acute care hospital	63	353					416

Supplemental Figure S1. UPMC system-level process for identifying, requesting, and reporting results of clusters investigated using whole genome sequencing.

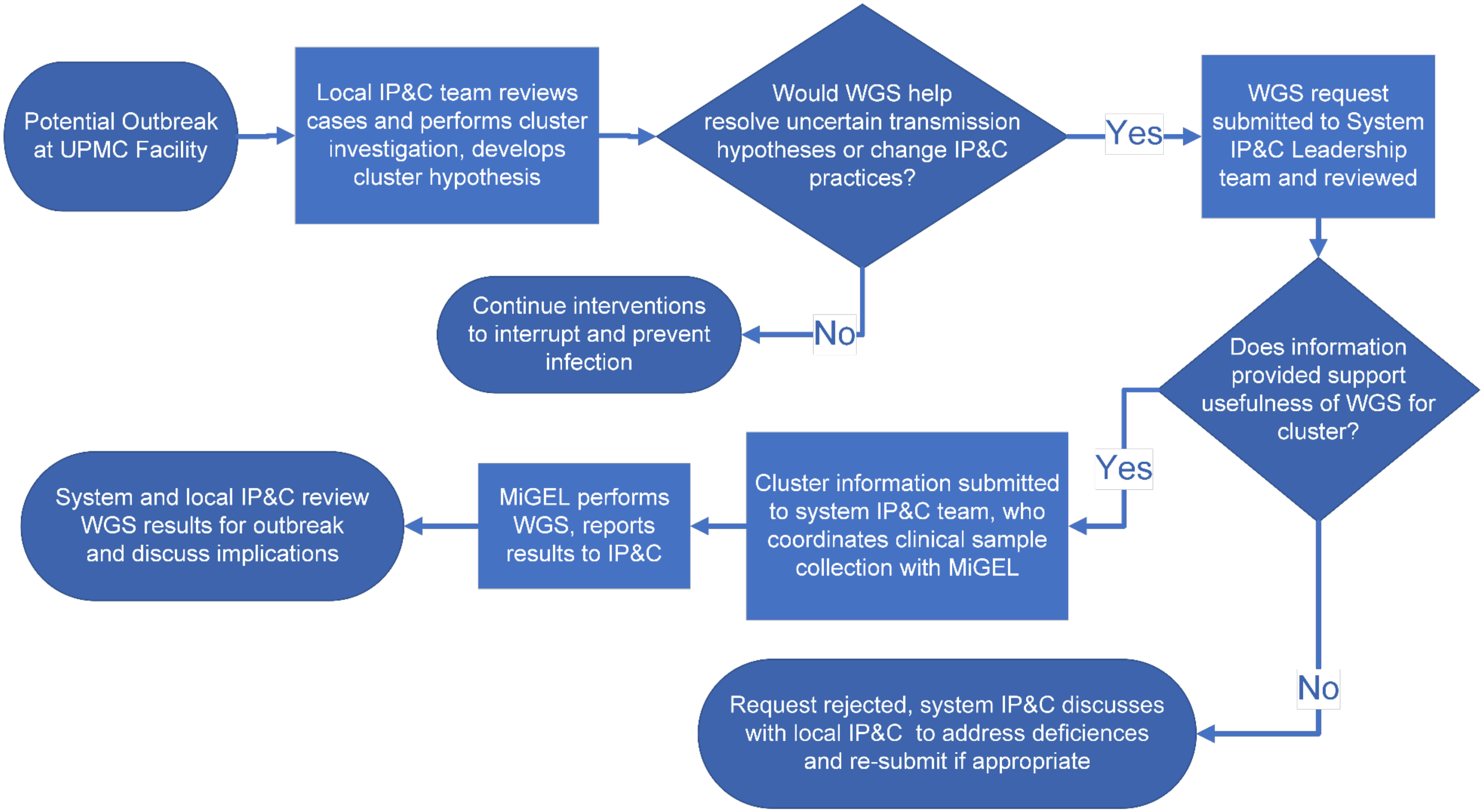

Note: IP&C, infection prevention & control; WGS, whole genome sequencing; MiGEL, Microbial Genomics Epidemiology Laboratory

Supplemental Figure S2. Structured interview tool to assess infection prevention & control teams' use of whole genome sequencing to support COVID-19 cluster investigation

Structured Interview Questionnaire

Participants: ____________________

UPMC Infection Prevention Group Location: ____________________

Outbreak Reviewed: ____________________

Questions regarding cluster investigation:

1. How/Why did your team consider using WGS to augment SARS-CoV-2 cluster investigation? (Long form answer)
2. Was/were there hypothesized transmission pathway(s) before the WGS request was placed? [Yes/No]
3. If there was more than one pathway/relationship predicted, please state the number of events suspected: (Numerical answer)
4. If there was more than one introduction predicted, please state the number of events suspected: (Numerical answer)
5. If there was no hypothesized transmission pathway, what other factors led to suspicion that patients in the outbreak could be related? (Numerical answer)
6. Was there a transmission visualization of the outbreak? [Yes/No]
    a. [If Yes] How many transmission visualizations were created for the outbreak? (Numerical answer)
        i. What factors motivated you to create a transmission visualization? (Long form answer)
        ii. What factors informed the transmission visualization(s) that you chose to create? (Long form answer)
    b. [If No] What factors would have motivated your team to create a transmission visualization for the outbreak? (Long form answer)
7. Did WGS cluster analysis of this outbreak help identify a definitive pathway(s)? [Yes/No]
    a. [if Y] Was that one of your hypothesized pathways? [Yes/No]
8. Did the WGS information change your understanding of the SARS-CoV-2 Nosocomial transmission during the outbreak in question, and in what ways did it change that understanding?? (Long form answer)
9. Did the WGS information from this cluster change your interventions to interrupt SARS-CoV-2 transmission at the time you received it? Describe these changes, and if no changes made, explain rationale? (Long form answer)

Note: WGS, whole genome sequencing.

Supplemental Table S2. Information cascade from cases suspected in healthcare-associated COVID-19 clusters to isolates with whole genome sequencing available

Information cascade	Patients		Healthcare workers		Total	
	No.	(%)	No.	(%)	No.	(%)
Cluster cases identified by infection prevention & control	132		94		226	
Clinical specimen not available	41	(31)	38	(40)	79	(35)
Specimen failed quality control for whole genome sequencing	19	(14)	12	(13)	31	(14)
Genomes available for whole genome sequencing analysis	72	(55)	44	(47)	116	(51)
Isolates genetically related* by whole genome sequencing to ≥1 isolate in the cluster	60	(83)	31	(70)	94	(81)

** Genetic relatedness defined by ≤2 single nucleotide polymorphisms*

Supplemental Table S3: Sequence information for SARS-CoV-2 genomes included in this study, including the Isolate ID, collection date, Pangolin lineage and Global Initiative on Sharing All Influenza Data (GISAID) ID.

Isolate ID	Collection date	Pangolin Lineage	GISAID ID
NCOV00329	5/15/2020	B.1	EPI_ISL_682003
NCOV00331	5/19/2020	B.1	EPI_ISL_682004
NCOV00396	6/23/2020	B.1.369	EPI_ISL_682005
NCOV00417	6/27/2020	B.1.369	EPI_ISL_682015
NCOV00418	6/28/2020	B.1.369	EPI_ISL_682016
NCOV00422	6/29/2020	B.1.369	EPI_ISL_682018
NCOV00442	7/22/2020	B.1.1.447	EPI_ISL_681930
NCOV00443	7/24/2020	B.1.1.447	EPI_ISL_681931
NCOV00444	7/24/2020	B.1.1.447	EPI_ISL_681932
NCOV00445	7/25/2020	B.1.1.447	EPI_ISL_681933
NCOV00446	7/24/2020	B.1.1.447	EPI_ISL_681934
NCOV00447	7/24/2020	B.1.1.447	EPI_ISL_681935
NCOV00700	10/6/2020	B.1.240	EPI_ISL_682031
NCOV00701	10/6/2020	B.1.240	EPI_ISL_682032
NCOV00702	10/7/2020	B.1.240	EPI_ISL_682033
NCOV00712	10/8/2020	B.1.240	EPI_ISL_682039
NCOV00713	10/9/2020	B.1.240	EPI_ISL_682040
NCOV00714	10/13/2020	B.1.240	EPI_ISL_682041
NCOV00763	10/19/2020	B.1.2	EPI_ISL_682050
NCOV00764	10/12/2020	B.1.2	EPI_ISL_682051
NCOV00765	10/19/2020	B.1.2	EPI_ISL_682052
NCOV00767	10/18/2020	B.1.2	EPI_ISL_682053
NCOV00768	10/18/2020	B.1.2	EPI_ISL_682054
NCOV00949	11/12/2020	B.1.2	EPI_ISL_853314
NCOV00954	2020-XX-XX	B.1.2	EPI_ISL_853316
NCOV00960	11/11/2020	B.1.2	EPI_ISL_853317
NCOV01032	11/24/2020	B.1.2	EPI_ISL_853327
NCOV01034	11/29/2020	B.1.2	EPI_ISL_853328
NCOV01086	11/19/2020	B.1.2	EPI_ISL_853329
NCOV01087	11/30/2020	B.1.2	EPI_ISL_853330
NCOV01088	12/3/2020	B.1.2	EPI_ISL_853331
NCOV01089	11/19/2020	B.1.2	EPI_ISL_853332
NCOV01090	11/30/2020	B.1.2	EPI_ISL_853333
NCOV01092	11/30/2020	B.1.2	EPI_ISL_853334
NCOV01093	11/24/2020	B.1.2	EPI_ISL_853335
NCOV01094	11/20/2020	B.1.2	EPI_ISL_853336

NCOV01096	12/2/2020	B.1.2	EPI_ISL_853337
NCOV01097	12/3/2020	B.1.2	EPI_ISL_853338
NCOV01147	12/1/2020	B.1.2	EPI_ISL_853339
NCOV01148	12/2/2020	B.1.2	EPI_ISL_853340
NCOV01149	12/8/2020	B.1.2	EPI_ISL_853341
NCOV01150	12/9/2020	B.1.2	EPI_ISL_853342
NCOV01153	12/10/2020	B.1.2	EPI_ISL_853343
NCOV01155	12/12/2020	B.1.2	EPI_ISL_853344
NCOV01157	12/14/2020	B.1.2	EPI_ISL_853345
NCOV01267	12/21/2020	B.1.311	EPI_ISL_853353
NCOV01268	12/19/2020	B.1.311	EPI_ISL_853354
NCOV01269	12/21/2020	B.1.2	EPI_ISL_853355
NCOV01270	12/20/2020	B.1	EPI_ISL_853356
NCOV01271	12/23/2020	B.1.2	EPI_ISL_853357
NCOV01272	12/23/2020	B.1.2	EPI_ISL_853358
NCOV01273	12/26/2020	B.1.311	EPI_ISL_853359
NCOV01274	12/21/2020	B.1.311	EPI_ISL_853360
NCOV01275	12/21/2020	B.1.311	EPI_ISL_853361
NCOV01276	12/23/2020	B.1.311	EPI_ISL_853362
NCOV01277	12/26/2020	B.1.2	EPI_ISL_17956673
NCOV01278	12/18/2020	B.1.311	EPI_ISL_853363
NCOV01279	12/19/2020	B.1.311	EPI_ISL_853364
NCOV01280	12/23/2020	B.1.2	EPI_ISL_853365
NCOV01281	12/23/2020	B.1.311	EPI_ISL_853366
NCOV01282	12/21/2020	B.1.311	EPI_ISL_853367
NCOV01284	12/26/2020	B.1.311	EPI_ISL_853368
NCOV01285	12/21/2020	B.1.2	EPI_ISL_853369
NCOV01286	12/20/2020	B.1.2	EPI_ISL_853370
NCOV01287	12/23/2020	B.1.2	EPI_ISL_853371
NCOV01288	12/18/2020	B.1.243	EPI_ISL_853372
NCOV01289	12/28/2020	B.1.311	EPI_ISL_853373
NCOV01290	12/27/2020	B.1.311	EPI_ISL_853374
NCOV01546	1/26/2021	B.1.1.222	EPI_ISL_1137192
NCOV01547	1/29/2021	B.1.1.222	EPI_ISL_1137191
NCOV01548	1/30/2021	B.1.1.222	EPI_ISL_1137189
NCOV01549	1/31/2021	B.1.1.222	EPI_ISL_1137187
NCOV01550	1/30/2021	B.1.1.222	EPI_ISL_1137190
NCOV01551	1/31/2021	B.1.1.222	EPI_ISL_1137186
NCOV01552	1/31/2021	B.1.1.222	EPI_ISL_1137188
NCOV01553	1/26/2021	B.1.243	EPI_ISL_1137182

NCOV01554	2/1/2021	B.1.243	EPI_ISL_1137183
NCOV02163	4/5/2021	B.1.1.7	EPI_ISL_1732496
NCOV02164	4/6/2021	B.1.1.7	EPI_ISL_1732497
NCOV02165	4/1/2021	B.1.1.7	EPI_ISL_1732498
NCOV02166	4/1/2021	B.1.1.7	EPI_ISL_1732499
NCOV02167	4/1/2021	B.1.2	EPI_ISL_1732500
NCOV02585	7/20/2021	AY.25	EPI_ISL_3341927
NCOV02586	7/20/2021	AY.25	EPI_ISL_3341928
NCOV02587	7/20/2021	AY.25	EPI_ISL_3341929
NCOV02588	7/20/2021	AY.25	EPI_ISL_3341930
NCOV02589	7/29/2021	AY.25	EPI_ISL_3341931
NCOV02590	7/23/2021	AY.25	EPI_ISL_3341932
NCOV02593	8/4/2021	AY.25	EPI_ISL_5327589
NCOV02597	8/2/2021	AY.25	EPI_ISL_5327543
NCOV02598	8/2/2021	AY.25	EPI_ISL_5327548
NCOV02599	8/2/2021	AY.25.1	EPI_ISL_17960472
NCOV02600	8/2/2021	AY.25	EPI_ISL_5327591
NCOV02601	8/1/2021	AY.25	EPI_ISL_5327549
NCOV02602	7/24/2021	AY.25	EPI_ISL_5327592
NCOV02604	8/8/2021	AY.25	EPI_ISL_5327550
NCOV02605	8/11/2021	AY.25	EPI_ISL_5327551
NCOV02606	8/6/2021	AY.25.1	EPI_ISL_5327552
NCOV02639	8/16/2021	AY.25	EPI_ISL_5327559
NCOV02640	8/11/2021	AY.25	EPI_ISL_5327534
NCOV02642	8/13/2021	AY.103	EPI_ISL_5327594
NCOV02643	8/18/2021	AY.25.1	EPI_ISL_5327539
NCOV02763	9/7/2021	AY.44	EPI_ISL_5327570
NCOV02766	9/10/2021	AY.44	EPI_ISL_5327572
NCOV02772	9/10/2021	AY.44	EPI_ISL_5327576
NCOV02773	9/15/2021	AY.44	EPI_ISL_5327577
NCOV02777	8/18/2021	AY.116.1	EPI_ISL_17956685
NCOV02778	10/4/2021	AY.39	EPI_ISL_17956684
NCOV02779	10/5/2021	AY.39	EPI_ISL_17956683
NCOV03302	6/7/2022	BA.2	EPI_ISL_17956677
NCOV03303	6/8/2022	BA.2	EPI_ISL_17956676
NCOV03304	6/7/2022	BA.2	EPI_ISL_17956678
NCOV03305	6/7/2022	BA.2	EPI_ISL_17956679
NCOV03307	6/6/2022	BA.2	EPI_ISL_17956680
NCOV03309	5/26/2022	BA.2	EPI_ISL_17956682
NCOV03310	6/2/2022	BA.2	EPI_ISL_17956681

Supplemental Figure S3. Pairwise single nucleotide polymorphism comparison for SARS-CoV-2 isolates identified in the healthcare-associated COVID-19 clusters

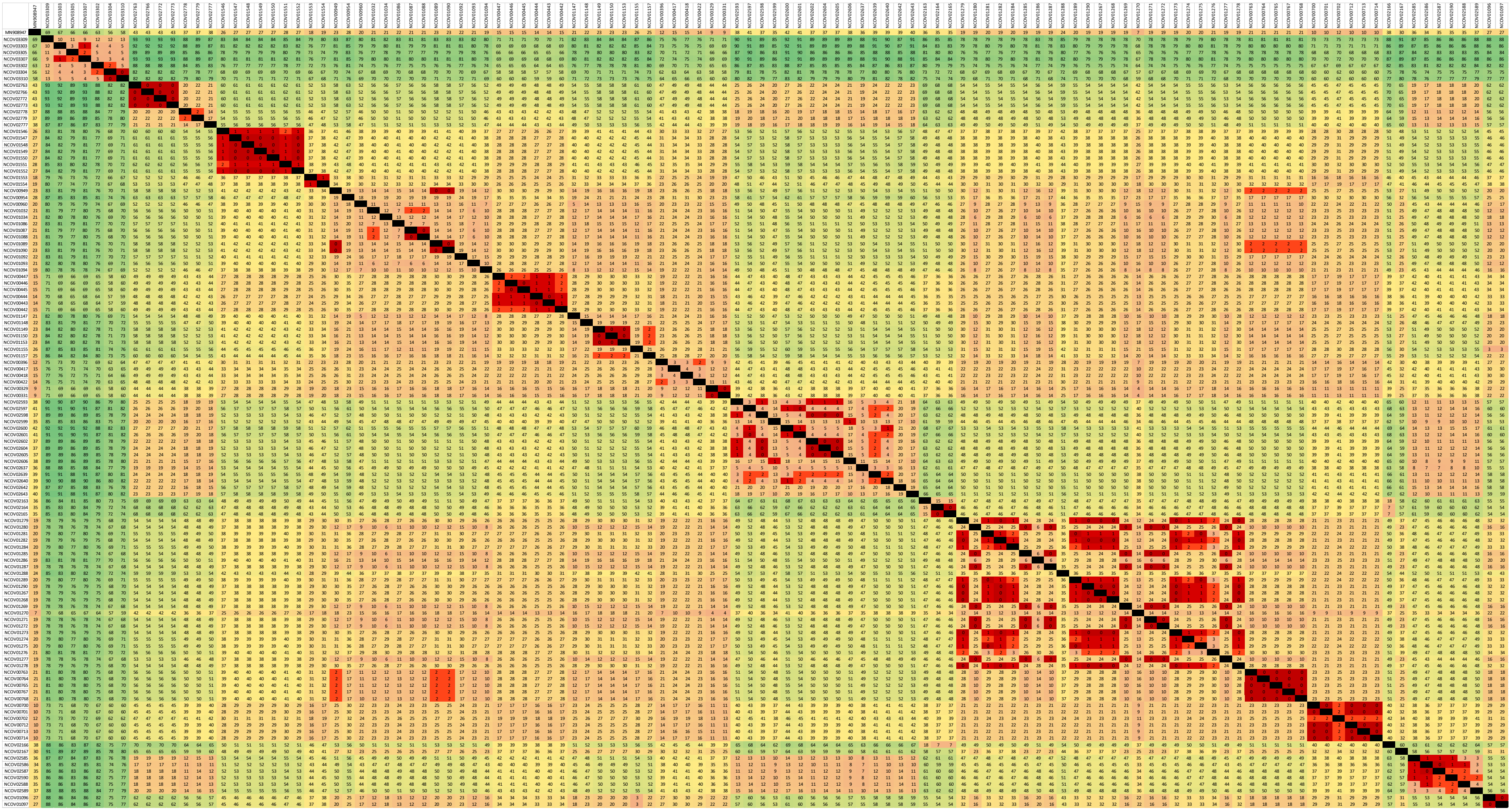

Colors indicate genetic relatedness: Green → red indicates more genetic relatedness. Darker red indicates ≤2 single nucleotide polymorphisms. First sequence in the column/row is the ancestral Wuhan-1 strain (MN908947) for comparison.

Supplemental Table S4a. Epidemiologic findings of COVID-19 cluster investigations in eight healthcare facilities

Cluster	Cluster designation	Case dates		Outbreak location type	Unique clusters	Epidemiologic (hypothesized) and WGS-supported transmission routes				
		First case	Last case			HCW to HCW	HCW to patient	Patient to HCW	Patient to patient	Unknown or none determined
1	H-1	5/11/2020	5/19/2020	Intensive care unit	1	WGS				EPI
2	G-1	6/18/2020	7/2/2020	Transitional care unit	1		EPI WGS*	WGS*		
3	F-1	7/22/2020	7/27/2020	Inpatient psychiatric ward	1		EPI WGS		WGS	
4	B-1	10/4/2020	10/14/2020	Inpatient rehabilitation unit	1	EPI WGS	EPI	EPI	EPI	
5	B-2	10/12/2020	10/19/2020	Inpatient rehabilitation unit	1		EPI WGS		EPI WGS	
6^	A-1	11/11/2020	12/3/2020	Five medical/surgical units, two intensive care units	2		EPI WGS		EPI WGS	
6^	H-2	11/29/2020	11/29/2020	Medical/surgical unit	0		EPI WGS		EPI WGS	
7	E-1	11/23/2020	12/14/2020	Inpatient rehabilitation unit	1		WGS	WGS	EPI WGS	
8	C-1	11/29/2020	12/3/2020	Inpatient rehabilitation unit	1				WGS	EPI
9	B-3	12/18/2020	12/28/2020	Two inpatient rehabilitation units	2	EPI WGS	EPI WGS	WGS		
10	A-2	1/26/2021	2/1/2021	Medical/surgical unit	2		EPI		EPI WGS	
11	B-4	4/1/2021	4/6/2021	Inpatient rehabilitation unit	1		WGS	WGS		EPI
12	D-1	4/1/2021	4/1/2021	Medical/surgical unit	0		EPI		EPI	WGS
13	D-2	7/20/2021	7/29/2021	Medical/surgical unit and associated long term care unit	1			EPI	EPI WGS	

14	B-5	7/24/2021	8/18/2021	Surgical/oncology units	3			WGS	EPI<br>WGS	
15	A-3	8/18/2021	10/10/2021	Non-patient care area	1	EPI<br>WGS				
16	A-4	8/29/2021	9/15/2021	Inpatient rehabilitation and medical/surgical unit	1	EPI<br>WGS	EPI<br>WGS			
17	F-2	5/26/2022	6/8/2022	Inpatient psychiatric ward	2				EPI<br>WGS	

Note: EPI, epidemiological; HCW, healthcare worker; ICU, intensive care unit; NA, not applicable; WGS, whole genome sequencing.
** For these clusters, WGS did not definitively determine whether either or both potential transmission pathways occurred.*
^ Clusters A-1 and H-2 occurred at two hospitals with one IP&C team, evaluated as a single cluster (6).

Supplemental Table S4b. Epidemiologic findings of COVID-19 cluster investigations in eight healthcare facilities

Cluster	Cluster designation	Outbreak Description<br>*Interventions*
1	H-1	Limited retrospective information available regarding outbreak due to IP&C staff attrition. We noted that several HCW in a single intensive care unit became symptomatic within a two week period. There were potentially multiple SARS-CoV-2 introductions into the cluster by HCW with positive outside contacts.
2	G-1	Outbreak started on a transitional care unit where multiple patients and HCW became positive within a two-week period. IP&C personnel were concerned that the outbreak would require unit closure if the cases were related. WGS was requested since results could inform the extent of cluster relatedness and assist in decision making. WGS results suggested spread between patients and HCW, but had no impact on unit closure as local IP&C team used other factors (not specified) to make that decision.<br>*Unit closed to limit spread, but no impact of WGS on the decision to make this intervention*
3	F-1	A HCW and a patient became symptomatic and positive on the same day on an inpatient psychiatric unit. Within five days, there were other symptomatic cases. The IP&C team suspected SARS-CoV-2 introduction could be from the positive HCW or from other patients (patient to patient or patient to HCW). HCW were staffed on multiple units and the transmission pattern was not completely clear due to the nature of the unit. WGS results suggested HCW was likely the source, followed by patient-patient transmission.<br>*IP&C team educated HCW groups that covered the affected units with prevention goals, considered increased ventilation in certain units, and changed the assessment of symptoms to help better identify potential outbreaks*
4	B-1	Outbreak started on an inpatient rehab unit with positive symptomatic patient being detected (requested specimen failed WGS). This prompted screening of all patients in double rooms on the unit, however, screening results were negative. Four days later, multiple HCWs and a patient tested positive, and there were more cases that followed. All of the HCWs confirmed to be related, however none of the patient genomes passed WGS QC.<br>*After strict monitoring of infection prevention practices and placing all patients on droplet/contact precautions, no further cases were detected on this unit.*
5	B-2	Outbreak began with an symptomatic patient at a rehabilitation unit, followed by another positive patient and three positive HCWs on the unit within five days, prompting IP&C team to perform asymptomatic screening of patients and HCWs. Four out of five patients in this cluster had close geographic relationships in a wing of the unit, and at least two were roommates, although suspected index patient was not geographically close. All patients also had therapy sessions in a common gym, where masking was difficult, due to the patient's previously existing neurologic injuries.<br>*IP&C team reinforced existing practices, such as ending common use of the rehabilitation gym for a short period of time, and placing all patients in contact/droplet precautions, which was sufficient to end the outbreak.*
6	A-1<br>H-2	Local IP&C team suspected multiple potential outbreaks due to the number of healthcare onset cases throughout Hospital A in a short period of time, raising concerns that there was significant asymptomatic transmission propagating in the facility. Contact tracing helped to identify potentially related cases that were added to the investigation. There was concern for additional spread in Hospital H (covered by same IP&C team) with possible HCW crossover between facilities prompting the addition of several Hospital H cases. Multiple routes of transmission were suspected, including support staff to patient, patient to patient, and HCW to patient. Genetic data indicated there were two patients with geographic proximity that could have shared common HCWs; however, one patient in each cluster had no epidemiologic connections readily identified with the others.<br>*Educated certain units. Reinforcing masking, hand hygiene, promoting unit huddles. This message was perceived as being concrete information that precautions were necessary, and this was "more comprehensible" to staff.*
7	E-1	An inpatient rehabilitation unit noted a large increase in the number of positive hospital-onset cases identified by the IP&C team. The IP&C team suspected SARS-CoV-2 was being introduced by support staff, and then spreading among patients (support staff to patient, then patient to patient). Several HCWs tested positive after the outbreak was suspected. WGS indicated that at least one HCW had been involved in transmission, which was an

		unexpected result by the IP&C team conducting the investigation, who felt protection measures had been adequately followed. Additionally, WGS indicated that multiple introductions of SARS-CoV-2 into the clinical environment were likely. *The IP&C team changed patient gym layout, ensuring adequate distances between patients and adequate labelling of zones for conduction of activities. Retroactively conducted interviews with patients, staff and support persons (visitors) to identify opportunities for improvements in process. Reviewed internal communications, able to establish better communications and information sharing within hospital to investigate future outbreaks better.*
8	C-1	An asymptomatic patient case followed by a symptomatic patient case were detected during routine screening on an inpatient rehabilitation unit. There was concern by IP&C team that a symptomatic positive HCW had introduced SARS-CoV-2 or the asymptomatic case had transmitted the virus to a patient staying in the room next door to the index case. Two other cases were suspected to be a related pair based on symptoms and timing, but the viral isolates did not pass WGS quality control criteria. WGS did confirm the suspicions of the IP&C team that the first two patients were related.
9	B-3	An outbreak on two inpatient rehabilitation units located on the same floor with shared HCW was detected. These units were locked, so patient-patient transmission between the units was considered unlikely; although, intra-unit transmission was possible. As suspected, WGS confirmed that the transmission between the two units was unlikely. A high degree of intra-unit transmission was found between patients and HCWs. Although the directionality could not be determined, IP&C suspected transmission between HCW to HCW and/or HCW to patient.
10	A-2	Two acute care medicine units with temporally-linked clusters of COVID-19 disease were noted had an occasional shared HCW and patients with significant risk for poor COVID-19 outcomes (post-thoracic transplant, severe cardiac and lung disease, including LVAD patients). The exact reason for the WGS request was limited by the IP&C team member leaving the system and lack of records. WGS found evidence of transmission within units, but not between units.
11	B-4	Inpatient rehabilitation unit saw multiple positive patients following a similar pattern as the other inpatient rehabilitation units in Hospital B during previous outbreaks (B 1-3). The IP&C team hypothesized one index case, but then multiple HCWs and patients became positive, which prompted a WGS request. There was a concern for HCW-patient transmission and introduction from support staff to patients. WGS determined transmission from HCW to HCW or patient to HCW, but not all positive cases were sequenced, making attribution difficult.
12	D-1	Concern on an acute medical/surgical unit of viral spread, with transmission route suspicions of patient to patient or HCW to patient. Local IP&C team requested WGS, to determine if transmission could have been patient-patient, given the fact that one of the positive patients was exposed multiple times within the potential incubation period. Index symptomatic patient was not tested on admission, but may have been symptomatic from COVID-19 and propagating this outbreak. WGS demonstrated that hospital roommates did not have the same source of infection, although a second possible source was identified, but viral isolate for this person was unavailable for WGS. *WGS results helped local IP&C communicate information about the isolation of roommates of positive COVID-19 patients, which had been less strictly monitored prior to this information being available.*
13	D-2	Outbreak in both an inpatient medical/surgical unit and long-term care center associated with Hospital D. Two potential index cases were found on two units of the long-term care facility, followed by several other residents of long-term care facility. One of the index cases recently transferred from an inpatient medical/surgical unit at the facility, leading to the concern that there could have been transmission during inpatient stays during the incubation/infection window. Several patients were included from the acute inpatient facility, where at least one index case was admitted in an overlapping time frame. Furthermore, the inpatient medical-surgical cases had close genetic relatedness.
14	B-5	Large single unit outbreak on a surgical and oncology patient floor. Multiple patients were discharged and then readmitted with COVID-19 within possible windows of exposure from their inpatient stay. WGS was requested to determine the degree to which the outbreak was related as the IP&C team suspected there was patient to patient transmission between hospital roommates, which was not observed in earlier outbreaks within the facility. Additionally, there was concern that two smaller clusters within the unit with stronger within-cluster spatiotemporal relationships were connected by common patients. A different cluster linkage method (average instead of single) was used, and found that there were three separate outbreaks with <2 SNPs between cases. One individual in the later onset subcluster (medium blue in Figure 2) had a three SNP difference from individuals in the second large subcluster (N=5 in both, see dark blue in Figure 2).

15	A-3	Multiple HCW cases were detected in a hospital department that was not patient facing. There was concern that a common area and/or equipment being used by multiple HCW in that department operated in close proximity for prolonged periods could have contributed to the spread. The related cases had no temporal overlap in the shared space, which was not initially suspected by the IP&C team investigating the cluster, leading to the concern for silent transmission.<br>*The department changed how employees utilized the common environment to try to better limit transmission, improved cleaning practices, and asked employees to take separate lunches.*
16	A-4	Multiple patients and HCWs tested positive in the same inpatient rehabilitation unit and medicine/surgical unit on the same floor. WGS determined there was a connection between the units, and thus, the local IP&C team re-investigated the outbreak with interviews. It was determined that one of the positive HCWs had provided assistance on both of the units that included the positive patients. This information was unavailable during the initial investigation, and local IP&C determined WGS helped them make this connection.<br>*The local IP&C team conducted education and nursing huddles, presenting this information to teams. No further clusters were observed on one of the involved units.*
17	F-2	Patients from an inpatient psychiatric unit were suspected to be related and contained in a single, large transmission cluster. An index case was identified that could have transmitted to all of the other patients. This unit was locked, but the patients had freedom of movement, and basic measures like social distancing and increased ventilation were present. WGS was performed but unexpectedly showed multiple introductions (at least three) to the unit, where two of these introductions involved multiple patients, none of whom were related to suspected index case.<br>*The local IP&C team felt confident that their actions prevented spread by this index patient, and reinforced certain aspects of these such actions with staff. This allowed some de-escalation of certain restrictions while maintaining others.*

Note: IP&C, Infection Prevention and Control; HCW, healthcare worker; WGS, whole genome sequencing; LVAD, Left Ventricular Assist Device; SNP, single nucleotide polymorphism.

Supplemental Figures S4a-S4c: Examples of data visualizations used to hypothesize transmission pathways in COVID-19 cluster investigations [all visualizations altered only for anonymization]

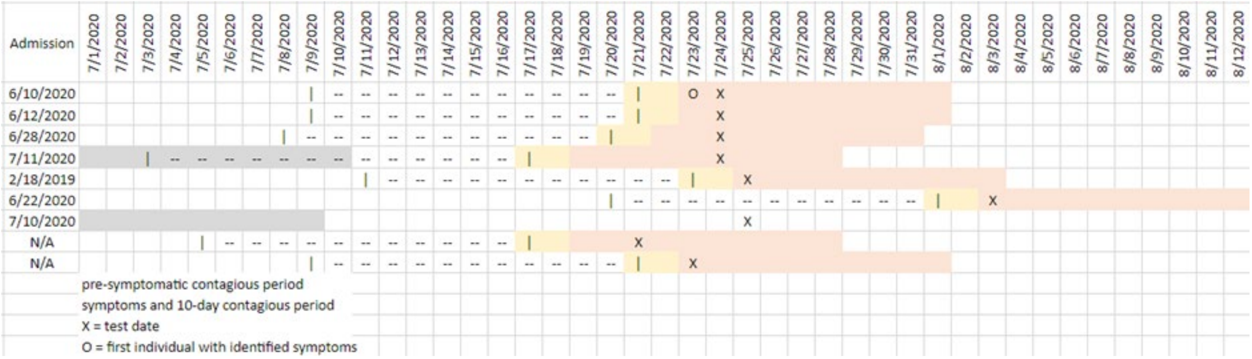

S4a. Example (cluster 3) of a transmission visualization using Gantt chart

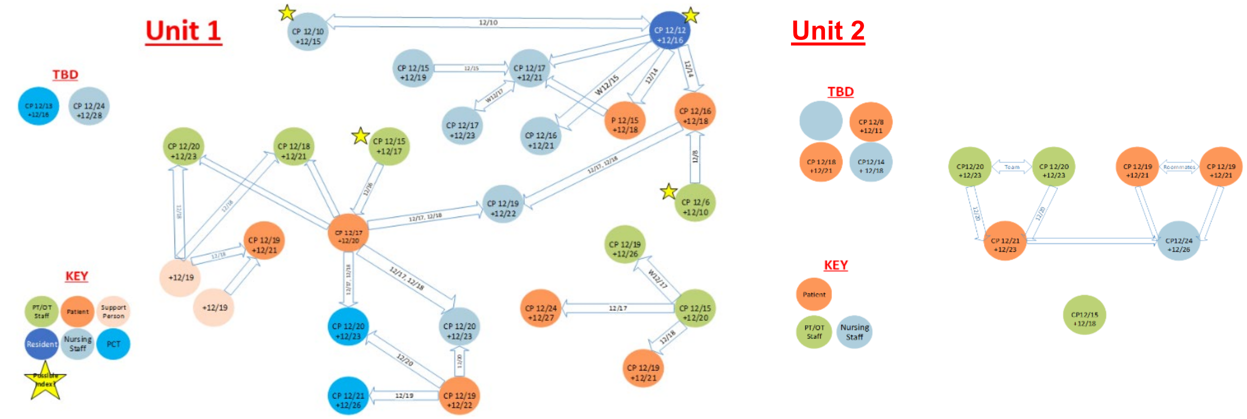

S4b. Example (cluster 9) of cluster visualizations (Spider charts) for two units used to guide transmission hypotheses for two clusters

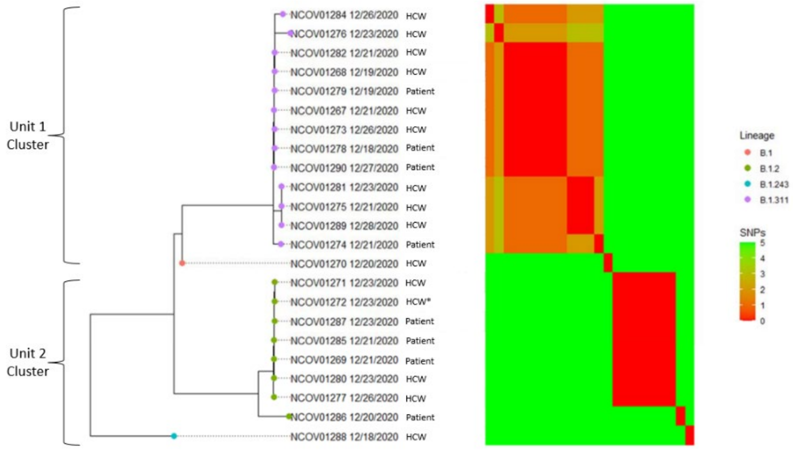

S4c. Example (cluster 9) of a heat map included in the WGS reports provided to the local infection prevention and control teams, with annotations, to aid in their cluster investigation. Bright red indicates close genetic relatedness (0-2 SNPS)
